# Enhancement of pomalidomide anti-tumor response with ACY-241, a selective HDAC6 inhibitor

**DOI:** 10.1371/journal.pone.0173507

**Published:** 2017-03-06

**Authors:** Brian J. North, Ingrid Almeciga-Pinto, David Tamang, Min Yang, Simon S. Jones, Steven N. Quayle

**Affiliations:** Acetylon Pharmaceuticals, Boston, Massachusetts, United States of America; University of Navarra, SPAIN

## Abstract

Thalidomide-based Immunomodulatory Drugs (IMiDs^®^), including lenalidomide and pomalidomide, are effective therapeutics for multiple myeloma. These agents have been approved with, or are under clinical development with, other targeted therapies including proteasome inhibitors, αCD38 monoclonal antibodies, as well as histone deacetylase (HDAC) inhibitors for combination therapy. HDAC inhibitors broadly targeting Class I and IIb HDACs have shown potent preclinical efficacy but have frequently demonstrated an undesirable safety profile in combination therapy approaches in clinical studies. Therefore, development of more selective HDAC inhibitors could provide enhanced efficacy with reduced side effects in combination with IMiDs^®^ for the treatment of B-cell malignancies, including multiple myeloma. Here, the second generation selective HDAC6 inhibitor citarinostat (ACY-241), with a more favorable safety profile than non-selective pan-HDAC inhibitors, is shown to synergize with pomalidomide in *in vitro* assays through promoting greater apoptosis and cell cycle arrest. Furthermore, utilizing a multiple myeloma *in vivo* murine xenograft model, combination treatment with pomalidomide and ACY-241 leads to increased tumor growth inhibition. At the molecular level, combination treatment with ACY-241 and pomalidomide leads to greater suppression of the pro-survival factors survivin, Myc, and IRF4. The results presented here demonstrate synergy between pomalidomide and ACY-241 in both *in vitro* and *in vivo* preclinical models, providing further impetus for clinical development of ACY-241 for use in combination with IMiDs for patients with multiple myeloma and potentially other B-cell malignancies.

## Introduction

While a variety of effective therapeutic options exist for patients with multiple myeloma (MM) including the immunomodulatory drugs (IMiDs^®^) thalidomide, lenalidomide and pomalidomide, a large number of patients remain refractory to, or undergo relapse to, IMiD treatment [1–3]. Thus, development of further combinations with these standard of care agents could enhance patient outcome [2]. IMiDs function by binding to the E3-ubiquitin ligase Cereblon and redirecting its activity towards the transcription factors IKZF1 (Ikaros) and IKZF3 (Aiolos) to drive their ubiquitination and subsequent proteasome-mediated degradation [4–8]. Depletion of these transcription factors in MM cell lines leads to inhibition of tumor cell growth, confirming the role of IMiD-mediated degradation of IKZF1 and IKZF3 on reducing myeloma cell proliferation [7]. Additionally, suppression of cellular proliferation by IMiDs is regulated by reduced expression of Myc and IRF4, factors which are frequently upregulated in MM patients and are established genetic dependencies [9–12]. Therefore, utilizing agents that further target Myc and IRF4 in combination with IMiDs could provide additional clinical efficacy and enhanced patient outcomes.

Modifications to histone proteins, including acetylation, phosphorylation, methylation, and ubiquitination, play key roles in regulating gene expression in normal tissues and can be aberrantly regulated in a wide variety of disease contexts including cancer. Histone deacetylases (HDACs) are enzymes that catalyze the removal of acetyl moieties on lysine residues on protein substrates. In the context of histone proteins, deacetylation of lysine residues drives transcriptional changes through chromatin remodeling within gene regulatory elements [13]. Additionally, HDACs target many non-histone proteins to regulate their function and/or stability [13]. HDAC inhibitors have been developed for cancer therapy in a variety of both solid and hematological malignancies, and transcriptional profiling of MM suggest HDAC inhibitors may be an attractive therapeutic target for the treatment of MM [14–17]. Previous studies have elucidated pathways regulated by HDACs and counteracted by HDAC inhibitors in cancer, including PTEN/Akt/mTor, p53, p21, p27 as well as cyclin/Cdk complexes, which when inhibited lead to the enhancement of cell cycle arrest and apoptosis that is observed with HDAC inhibitors [15, 18, 19]. Treatment of cancer cell lines with HDAC inhibitors frequently also leads to the downregulation of Myc, thereby enhancing cell death in diverse cancer cell types [19–21]. Given that MM cells show addiction to Myc [10], these data suggest a mechanistic link by which HDAC inhibitors could enhance cytotoxicity of MM cells through regulation of Myc expression. Together, these findings support the rationale that treatment with HDAC inhibitors in combination with IMiDs could enhance anti-tumor activity, including in the MM setting [20, 22].

HDAC inhibitors are broadly segregated into two classes, those that inhibit both Class I (HDAC1-3 and 8) and IIb (HDAC6 and 10) enzymes and those that inhibit Class I enzymes only [23]. While the pan-HDAC inhibitors vorinostat, belinostat, romidepsin, and panobinostat have been approved by the FDA for treatment of T-cell lymphoma or MM, their clinical utility is frequently limited due to poor tolerability, particularly in combination settings [24–27]. Therefore, the identification of HDAC inhibitors with reduced Class I HDAC inhibition may provide similar therapeutic potential while mitigating adverse side effects. Ricolinostat (ACY-1215), the first-in-class HDAC6 selective inhibitor which is 10-15-fold selective for HDAC6 over HDAC1-3, has exhibited preliminary efficacy in early clinical trials with an acceptable safety profile in combination with lenalidomide and dexamethasone [28]. Here, we demonstrate that citarinostat (ACY-241), a second generation HDAC6 selective inhibitor, shows combination efficacy with IMiDs in both *in vitro* and *in vivo* models of MM. Combination treatment resulted in increased apoptosis as well as cell cycle arrest, coupled with decreased expression of pro-survival genes. These results support the rationale of the ongoing Phase 1a/b clinical trial (NCT02400242) exploring combination treatment of MM patients with ACY-241 plus pomalidomide and dexamethasone.

## Results

**Fig 1:**
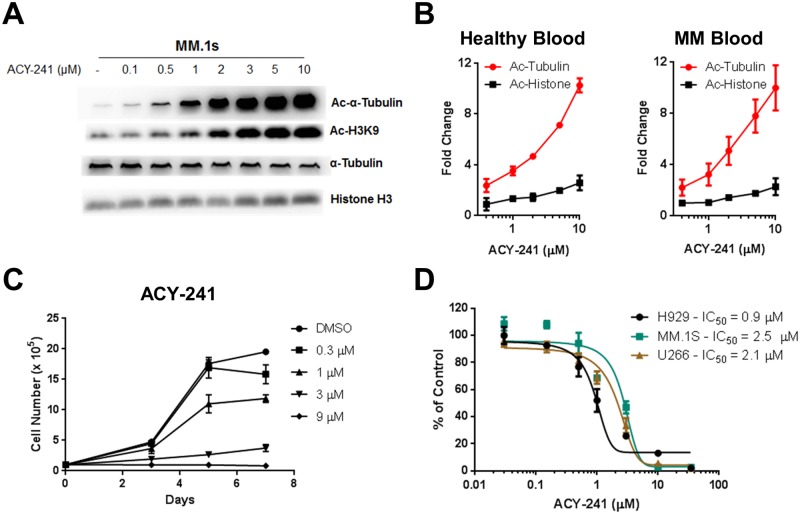
ACY-241 is a selective HDAC6 inhibitor that inhibits myeloma cell proliferation and survival. A) MM.1s cells were treated with ACY-241 for 18 hrs and lysates western blotted for Ac-α-Tubulin, Ac-Histone H3K9, total α-Tubulin and total histone H3. B) Blood from healthy donors and MM patients were treated for 18 hrs *ex vivo* with ACY-241 and stained with αCD3 and either Ac-α-tubulin or Ac-Histone H2BK5 along with isotype controls. CD3^+^ cells were gated followed by measuring MFI and normalizing against isotype and DMSO treated samples. C) H929 cells were treated with ACY-241 and live cells were counted following trypan blue staining. D) MM cell lines were treated with increasing concentrations of ACY-241 for 4 days and cell viability was measured using an MTS assay. The IC_50_ was determined for each cell line.

ACY-241 is a second generation HDAC6 selective inhibitor with 13 to 18-fold selectivity towards HDAC6 in comparison to HDAC1-3 [29]. To assess relative selectivity of this compound in MM cells, MM.1s cells were incubated with increasing concentrations of ACY-241 followed by assessment of lysine-40 acetylation (Ac) of α-Tubulin (an HDAC6 specific substrate) [30] and lysine-9 acetylation of Histone-H3 (H3K9; a Class I HDAC specific substrate) [31]. Acetylation of α-Tubulin began to increase at concentrations as low as 100–500 nM of ACY-241 whereas histone acetylation was observed to increase at 2 μM, confirming the HDAC selectivity profile of ACY-241 in MM cells (Fig 1A). Furthermore, utilizing a flow cytometry based assay developed to detect both acetylated α-Tubulin and acetylated Histone levels, we observed that *ex vivo* treatment of blood isolated from both healthy and myeloma patient donors with increasing concentrations of ACY-241 led to a greater increase in acetylated α-Tubulin levels relative to acetylation of Histones (Fig 1B). These findings are consistent with the demonstrated pharmacodynamic effects of the structurally related HDAC6 selective inhibitor ACY-1215 in MM patients [28]. Previously, we have shown that ACY-1215 reduces cellular proliferation and induces cell death in MM cells [32]. To directly compare the effect of these two distinct HDAC6 selective compounds *in vitro*, we performed cellular proliferation and viability analysis. H929 cells were treated with increasing concentrations of either ACY-241 or ACY-1215 and cellular proliferation was monitored. Both ACY-241 and ACY-1215 demonstrated similar dose-dependent suppression of H929 cell proliferation upon prolonged exposure (Fig 1C and S1A Fig). Furthermore, we performed cellular viability assays with increasing concentrations of ACY-241 and ACY-1215, and both showed similar IC_50_ values in H929 cells (S1B Fig), and ACY-241 similarly reduced the viability of the additional MM cell lines MM.1s and U266 (Fig 1D), as well as the MCL cell line Jeko-1 (S1C Fig). These results confirm that single agent ACY-241, similar to ACY-1215, is capable of reducing MM cell viability. Given its distinct structure and unique physiochemical properties, our further *in vitro* and *in vivo* analyses focused on ACY-241 to characterize its activity in preclinical models and support potential clinical testing.

**Fig 2:**
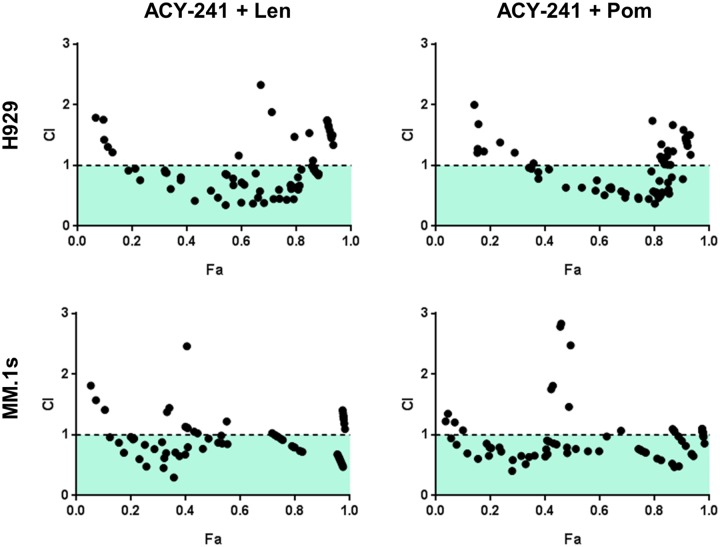
ACY-241 synergizes with lenalidomide and pomalidomide to suppress viability of cancer cells. H929 and MM.1s cells were treated with increasing concentrations of ACY-241 and either lenalidomide or pomalidomide in an escalating concentration matrix. Cells were incubated for 3 days followed by measuring cell viability by MTS assay. CI values were calculated, with a CI value <1 indicating synergistic activity of the combination over single agent treatment. Data shown is representative of three independent experiments in each cell line.

It was previously shown that the combination of HDAC inhibitors with IMiDs leads to enhanced cellular cytotoxicity [20]. Given this finding and our initial results demonstrating single agent anti-tumor activity of ACY-241, we assessed if ACY-241 enhanced the anti-proliferative effects of thalidomide-based IMiDs including lenalidomide and pomalidomide. To assess synergy of this combination, we performed MTS assays across a 96-point dose concentration matrix. Clear synergy was observed across a broad range of Fraction Affected (F_A_) values as indicated by Combination Index (CI) values less than 1 as calculated by the method of Chou-Talalay [33] (Fig 2). This synergistic reduction in viability was observed in both the H929 and MM.1s MM cell lines (Fig 2) and the Mantle Cell Lymphoma (MCL) cell line Jeko-1 (S2A Fig) when combining ACY-241 with either lenalidomide or pomalidomide. These results are very similar to those obtained when testing combinations of IMiDs with ACY-1215 (S2B Fig), confirming broad synergy of these selective HDAC inhibitors with the IMiD class of drugs in MM cell lines, and suggesting activity in other B-cell malignancies where IMiDs are active, such as MCL.

**Fig 3:**
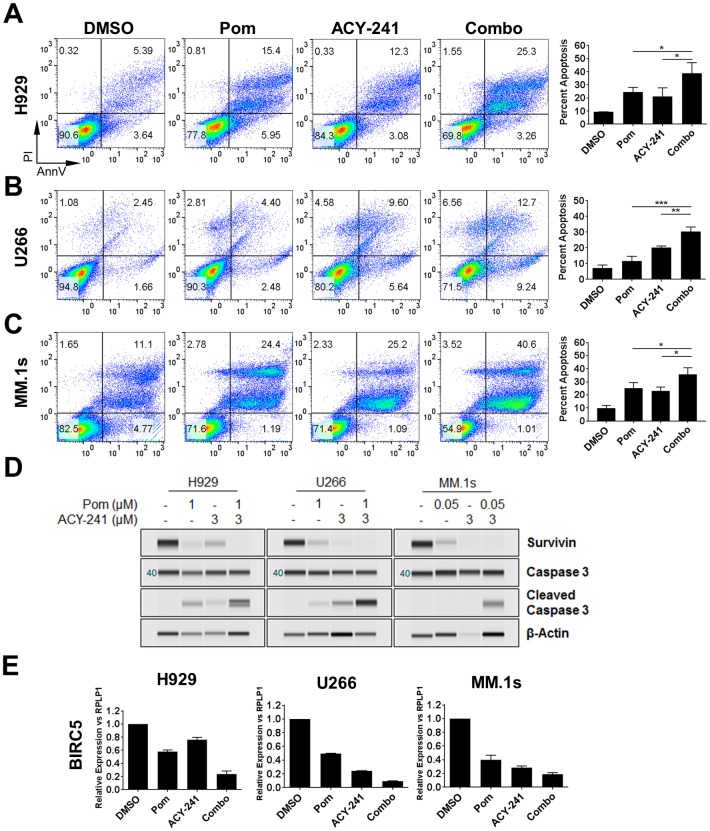
Combination treatment with ACY-241 and pomalidomide leads to increased apoptosis. H929 (A), U266 (B), and MM.1s (C) cells were treated with 1 μM or 0.05 μM pomalidomide or 3 μM ACY-241 alone or in combination for 4 days followed by staining for Annexin V/PI to measure apoptosis. Percent apoptosis was assessed by double positivity for Annexin V/PI. Representative flow dot plots are shown for each cell line (*left*) and the mean ± SD of three independent experiments is plotted (*right*). * *p* < 0.05, ** *p* < 0.01; *** *p* < 0.001. D) Cells treated as in A-C were harvested after 48 hours. Total protein was isolated and probed with antibodies for Survivin, Caspase 3, Cleaved Caspase 3, and β-Actin. Results are representative of at least 3 independent experiments for each antibody. E) Cells treated as in A-C were harvested after 48 hours. Total RNA was isolated and converted to cDNA followed by real-time PCR for *BIRC5* (survivin). Results were normalized to the housekeeping gene *RPLP1*, and the mean ± SD of three independent experiments is shown.

Given that both of these agents promote cell cycle arrest and cytotoxicity, we set out to elucidate the cellular mechanism(s) driving the combination benefit observed on viability. Three MM cell lines (H929, U266 and MM.1s) were treated with ACY-241 alone or in combination with pomalidomide followed by assessment of cellular apoptosis by measuring Annexin V surface staining and propidium iodide (PI) uptake. In each cell line we observed significantly increased apoptosis when both agents were treated in combination relative to either single agent (Fig 3A–3C). Interestingly, the efficacy of each single agent was largely cell line specific at the concentrations and time points measured, but enhanced apoptosis upon combination treatment was observed regardless of differences in single agent efficacy. For instance, in H929 and MM.1s cells, pomalidomide and ACY-241 had comparable effects on apoptosis (Fig 3A and 3C), whereas U266 cells were more susceptible to ACY-241 than to pomalidomide (Fig 3B). While these studies focused primarily on cellular models of MM, we observed similar results with regard to enhanced apoptosis in the Jeko-1 MCL cell line (S3A Fig). These results indicate that in each of the cell lines tested, treatment with ACY-241 and pomalidomide had a combination effect that was significantly greater than either single agent alone (Fig 3A–3C and S3A Fig), confirming that enhanced cytotoxicity contributes to the synergistic efficacy of these agents in cell viability assays.

Consistent with the described increase in apoptosis, we also observed in all cell lines a correlation between apoptosis and decreased survivin protein levels and increased cleavage of caspase 3 (Fig 3D and S3B Fig). Survivin is a member of the inhibitor of apoptosis (IAP) family of proteins that both inhibit apoptosis and regulate the cell cycle [34]. Survivin is upregulated in a wide variety of human cancers including MM, and survivin expression correlates with MM disease progression [35]. We further observed that downregulation of survivin correlated with decreased survivin (*BIRC5*) gene expression, both with single agent treatment and further suppression upon combination treatment (Fig 3E and S3C Fig). These data suggest that increased cytotoxicity through treatment with ACY-241 and pomalidomide is mediated in part through downregulation of the anti-apoptotic protein survivin.

**Fig 4:**
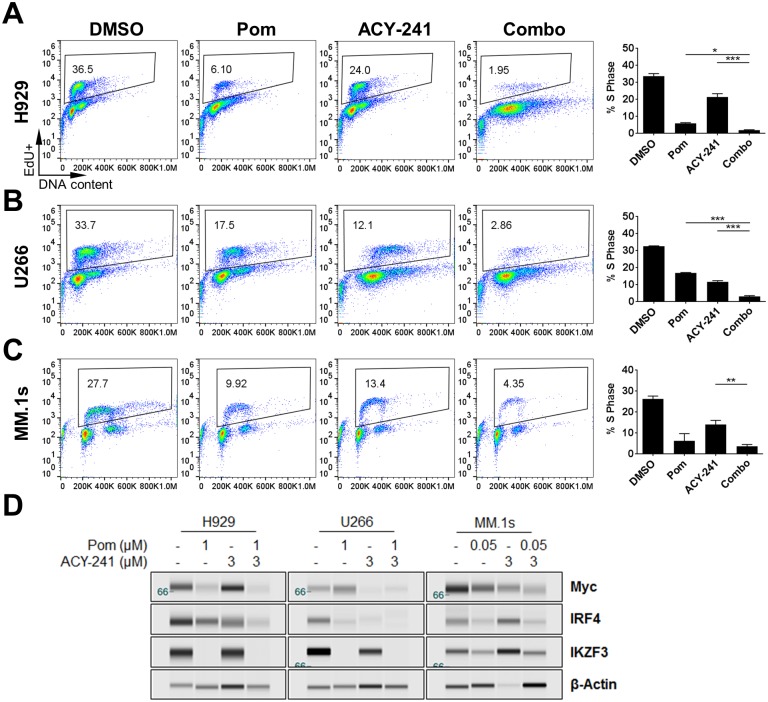
Combination treatment with ACY-241 and pomalidomide reduces S phase frequency. H929 (A), U266 (B), and MM.1s (C) cells were treated with 1 μM or 0.05 μM pomalidomide or 3 μM ACY-241 alone or in combination for 3 days followed by incubation for 1 hour with EdU and stained for EdU incorporation and FxCycle Far Red to measure S phase frequency. Percent cells in S phase was determined by gating EdU positive cells. Representative flow dot plots are shown for each cell line (*left*) and the mean ± SD or three independent experiments is plotted (*right*). * *p* < 0.05, ** *p* < 0.01; *** *p* < 0.001. D) Cells treated as in A-C were harvested after 48 hours. Total protein was isolated and probed with antibodies for Myc, IRF4, IKZF3 and β-Actin. Results are representative of at least 3 independent experiments for each antibody.

To determine if cell cycle arrest represents an additional mechanism driving the synergistic effects observed with ACY-241 and pomalidomide combination treatment, cell cycle profiles were assessed in each of four cell lines treated for 72 hours with ACY-241 or pomalidomide alone or in combination. DNA replication in S phase was monitored via incorporation of EdU into newly synthesized DNA. Utilizing percent cells in S phase as a readout for cell cycle arrest, we observed that treatment with ACY-241 or pomalidomide alone, or in combination, led to significant cell cycle arrest, albeit to varying degrees (Fig 4A–4C and S3D Fig). Interestingly, percent cells in S phase did not necessarily show a combination effect relative to both single agents in all cell lines assessed. For instance, in H929 and U266 cells, both pomalidomide and ACY-241 promoted cell cycle arrest as single agents and cell cycle progression was further suppressed following combination therapy (Fig 4A and 4B). However, in MM.1s and Jeko-1 cells, cell cycle arrest was not further reduced by combination treatment compared to treatment with single agent pomalidomide alone (Fig 4C and S3D Fig). In conclusion, treatment of each of these cell lines with the combination of ACY-241 and pomalidomide has a synergistic effect on viability by impacting both proliferation and cell death, though in some cell lines the induction of apoptosis by combination treatment may be the most significant contribution to improved efficacy of the combination.

The primary mechanism of action by which IMiDs promote cell cytotoxicity is via redirecting Cereblon-mediated ubiquitination and degradation of the transcription factors IKZF1 and IKZF3, resulting in changes in gene expression and cell death [4–8]. Analysis of cell lines treated with ACY-241 and pomalidomide either alone or in combination confirmed that pomalidomide treatment of each line reduced IKZF3 protein levels while not significantly affecting transcriptional regulation of *Cereblon* or *Ikaros* gene expression (Fig 4D and S3E and S4 Figs), consistent with previous reports [4–6]. Combination treatment also further downregulated Myc expression at the protein level, but not the mRNA level, suggesting that post-translational mechanisms may mediate Myc downregulation by the combination of these agents (Fig 4D and S3E and S4 Figs). In addition, IRF4 was downregulated at both the protein and mRNA level by pomalidomide and ACY-241 alone and in combination (Fig 4D and S3E and S4 Figs), suggesting enhanced suppression of IRF4 expression may also contribute to the observed combination efficacy. To extend the relevance of these findings, we observed that these pathways were altered in a similar pattern when cells were treated with ACY-241 in combination with lenalidomide (S5 Fig), indicating that combination efficacy was not pomalidomide specific but consistent across thalidomide-based IMiD molecules. These results are consistent with combination treatment of ACY-241 plus pomalidomide enhancing cytotoxicity via suppression of the Myc, IRF4 and survivin pro-survival pathways.

**Fig 5:**
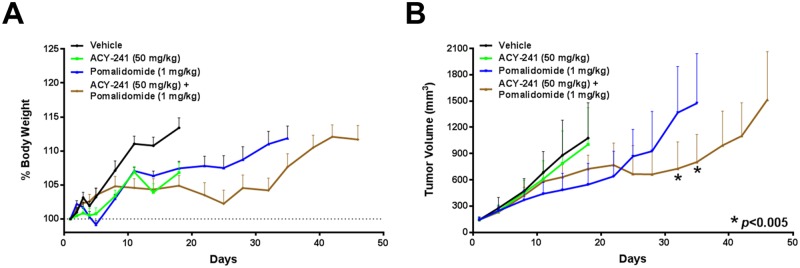
Combination treatment with ACY-241 and pomalidomide reduced tumor growth *in vivo*. A) Body weight was assessed throughout the period of treatment with vehicle, ACY-241, pomalidomide, or the combination. Graph shows mean relative body weight change ±SD. B) Enhanced suppression of H929 tumor xenograft growth after treatment with vehicle, ACY-241, pomalidomide, or the combination (n = 10 per treatment group). Graph shows mean tumor volume ±SD over the course of treatment. Each line is truncated when the first animal in each cohort was sacrificed due to humane or tumor volume endpoints. * *p* < 0.005 for combination treatment relative to pomalidomide alone.

To extend our findings with this combination to the *in vivo* setting, we assessed the efficacy of treating mice harboring H929 MM xenografts with ACY-241 and/or pomalidomide. Mice harboring palpable tumors were treated with either vehicle, ACY-241 (50 mg/kg QD), pomalidomide (1 mg/kg QD), or both agents in combination. Consistent with reduced toxicity of selective HDAC inhibitors, we did not observe any significant toxicity or adverse effects on the health of the animals as body weight increased while on treatment and no clinical signs were observed (Fig 5A). While ACY-241 did not significantly impact tumor growth as a single agent, pomalidomide treatment did suppress tumor growth relative to the vehicle treated group (Fig 5B). Importantly, combination treatment with ACY-241 plus pomalidomide resulted in significantly greater tumor growth inhibition relative to pomalidomide alone (Fig 5B). These results confirm that the combination benefit of ACY-241 and pomalidomide observed *in vitro* translates to combination efficacy *in vivo*.

## Discussion

Here we demonstrate that ACY-241, a second generation selective HDAC inhibitor, synergizes with the IMiD class of drugs to suppress proliferation and viability of tumor cells derived from MM. These results suggest the combination benefit largely derives from enhanced cytotoxicity through increasing cellular apoptosis, as well as cell cycle arrest in at least a subset of cell lines. At the molecular level, combination treatment resulted in greater suppression of Myc and IRF4 expression, as well as reduced expression of the anti-apoptotic protein survivin. Furthermore, we observed that this combination effect *in vitro* translated to significant combination efficacy on reducing tumor growth in an *in vivo* MM xenograft model. Together these results indicate that combining ACY-241 with IMiDs enhances anti-cancer efficacy in models of MM.

Our results demonstrate that ACY-241 treatment in combination with pomalidomide enhances apoptosis and cell cycle arrest compared to each agent alone. Interestingly, while each of the tested cell lines had a different sensitivity to each single agent, enhanced combination efficacy was observed in all cell lines tested. These results indicate that each compound enhances the cytotoxic effects of the partner agent, suggesting potentially broad applicability of this combination. The ability of ACY-241 to enhance response to IMiD treatment, even in lines that poorly respond to pomalidomide (such as U266), provide rationale for further assessment of combinations with ACY-241 to potentially resensitize IMiD-resistant MM models. Furthermore, our findings provide evidence of combination efficacy in the MCL cell line Jeko-1, suggesting potentially broader applicability of this combination across B cell malignancies. Future studies should explore additional models of other B cell malignancies in greater depth to determine if this combination acts through similar pathways as in models of MM.

Pomalidomide was previously shown to enhance Cereblon-mediated degradation of the transcription factors IKZF1 and IKZF3, which subsequently leads to suppression of the critical transcription factors Myc and IRF4 [9–12]. Non-selective HDAC inhibitors were also previously shown to downregulate Myc expression [36, 37], which has been shown to be further enhanced by IMiD treatment [20]. Consistent with these previous results, combination treatment of MM cells with pomalidomide and ACY-241 enhances suppression of both Myc and IRF4. Similar to Myc, the anti-apoptotic protein survivin is upregulated in a wide variety of human cancers including MM, and its expression correlates with MM disease progression [35]. Interestingly, we also demonstrate that the pro-survival protein survivin is significantly suppressed at both the mRNA and protein levels upon combination treatment compared to either agent alone. Furthermore, the response and degree of survivin suppression in each line correlated with the extent of apoptosis, suggesting that regulation of survivin may be a key determining factor driving cytotoxicity in response to the combination of these agents. Previous studies have demonstrated that HDAC inhibitors can suppress survivin [38, 39], which has been mechanistically linked to regulation of miR-203 in NSCLC tumor models [40], as well as to activation of AMP-activated protein kinase (AMPK) or the MAPK pathway (P38) in colon cancer models [41]. Our results indicate that combined regulation of Myc and survivin pathways may be key factors driving cellular cytotoxicity in MM cells in response to this combination treatment approach. Given that the doses of ACY-241 used in this study demonstrated pharmacodynamic regulation of both HDAC6 and Class I HDACs, future studies exploring the role(s) of specific HDAC isozymes in the regulation of Myc and survivin expression will be important to further define the mechanism of action of selective HDAC inhibitors in this combination in models of MM.

IMiDs have also been shown to modulate the immune system in addition to causing direct cytotoxicity of tumor cells through the regulation of IKZF1 and IKZF3 protein abundance [8]. Therefore, it would be of interest for future studies to assess the ability of selective HDAC inhibitors, including ACY-241, to enhance the immunomodulatory function(s) of IMiD molecules. In this study, we demonstrate that treatment with ACY-241 further suppressed the Myc and IRF4 pathways, as well as the pro-survival factor survivin, to promote cellular cytotoxic effects. Together these findings support the ongoing Phase 1 clinical trial of ACY-241 in combination with pomalidomide and dexamethasone for the treatment of MM patients (NCT02400242).

## Materials and methods

### Cell lines and reagents

Multiple myeloma cell lines MM.1s, H929, and U266, and the Mantle Cell Lymphoma cell line Jeko-1 were obtained from ATCC (Manassas, VA). MM.1s cells were cultured in Dulbecco's Modified Eagle's Medium (DMEM) supplemented with 10% fetal bovine serum (FBS), H929 cells were cultured in RPMI 1640 with 10% FBS, U266 cells were cultured in RPMI 1640 with 15% FBS, and Jeko-1 cells were cultured in RPMI 1640 with 20% FBS.

Ricolinostat (ACY-1215) and citarinostat (ACY-241) were synthesized by ChemPartner (Shanghai, China). Lenalidomide and pomalidomide were obtained from Selleck Chemicals (Houston, TX). All agents were dissolved in DMSO for *in vitro* use and stored at -20°C in single use aliquots.

### Detection of acetylated tubulin and histone in human blood by flow cytometry

Blood samples from healthy donors were collected at Research Blood Components, LLC. (Boston, MA). The collection of whole blood for research purposes was approved by New England IRB (NEIRB #04–144). Blood samples from multiple myeloma patients were collected at Bioreclimation, LLC. (Westbury, NY). The collection of patient whole blood was approved by Schulman IRB (IRB #201601934). All the participants provided their written informed consent to participate in this study, and blood was provided in sodium heparin tubes. 0.5 mL of whole blood was incubated with the indicated concentrations of ACY-241 at 37°C for 18 hrs. After incubation, 0.5 mL of 20% DMSO in PBS (v/v) was added, samples were mixed by inversion several times, and frozen in a slow freezing container. The frozen samples were thawed at 37°C and 200 μL of blood was aliquoted to tubes. An equal volume of 3.8% formaldehyde in PBS was added to each sample and incubated for 15 minutes at 37°C. Samples were washed three times with permeabilization buffer (0.5% BSA and 0.5% Triton X-100 in PBS) and incubated for 1 hour with primary antibodies for acetyl-α tubulin (Sigma), murine IgG control (Sigma), α-acetyl-histone H2BK5, (Cell Signaling), and rabbit control IgG (Cell Signaling). Samples were then washed three times with permeabilization buffer to remove excess primary antibody and stained with secondary antibodies Dylight488 goat anti-mouse IgG (KPL) and Dylight488 goat anti-rabbit IgG (KPL) for 15 minutes at 37°C. Samples were washed once with permeabilization buffer and once with PBS, then resuspended in 500 μL PBS and αCD3 antibody (eBioscience). Samples were allowed to stand for 15 minutes at room temperature and then assayed by flow cytometry. Data analysis began by gating on the CD3^+^ cells in FlowJo software (Treestar, Inc., Ashland, OR) and applying a median fluorescence intensity (MFI) statistic. Fold change was calculated by subtracting the control IgG MFI from the sample MFI and dividing the MFI of treated groups by the MFI of the DMSO treated control. The data illustrated are an aggregate of three different donor or patient samples (n = 3).

### Immunoblotting

Cells cultured in the presence of ACY-241 and/or pomalidomide were washed and lysed using radio-immunoprecipitation assay (RIPA) lysis buffer with HALT complete protease inhibitor cocktail (Thermo Scientific).

For western blot analysis lysates were kept at 4°C and sonicated in a Diagenode Bioruptor (Denville, NJ) on the “high” setting for five 30-second cycles on, five cycles off to ensure complete membrane disruption, and then centrifuged at 14,000 rpm for 10 minutes to clarify. Equal amounts of protein were loaded and size separated using the Life Technologies Bolt system with manufacturer recommended reagents and probed using antibodies against acetyl-α-tubulin (Sigma), acetyl-histone H3K9 (Millipore), α-Tubulin (Sigma), and histone-H3, pantropic (Millipore). Antigen-antibody complexes were detected using secondary antibodies conjugated with horseradish peroxide (HRP) and visualized on a Syngene Gbox (Frederick, MD).

For WES analysis, lysates were separated by an automated capillary-based electrophoresis system (WES, ProteinSimple, San Jose, CA). All procedures were performed according to the manufacturer’s recommendations using the supplied reagents. Briefly, after determination of protein concentration of each lysate, 0.4 mg of total protein (4 μL) was mixed with 1 μL of 5X fluorescent master mix and heated at 95°C for 5 min. The samples, blocking reagent, wash buffer, primary antibodies, secondary antibodies, and chemiluminescent substrate were dispensed into designated wells in the manufacturer provided microplate. Primary antibodies used are as follows: Survivin (Novus Biologicals), Caspase 3, c-Myc, IRF4, (Cell Signaling), IKZF3 (abcam), and β-Actin (Sigma). Following plate loading the separation and immunodetection was performed automatically using default settings. The resulting data were analyzed using Compass software (ProteinSimple).

### Cell proliferation

Cells were plated on day 0 in 12 well plates at a concentration of 100,000 cells per mL in 1 mL total volume. Cells were treated with the indicated concentrations of ACY-1215 or ACY-241 in triplicate. On day 0, 3, 5, and 7, cells in each well were mixed to resuspend and 20 μL were removed and mixed 1:1 with Trypan Blue. Viable cells, determined by exclusion of Trypan Blue, were counted. Triplicate values were averaged and SEM calculated using Excel (Microsoft) and graphed with Prism software (GraphPad).

### Cell proliferation assay

The growth inhibitory effect of ACY-241 or ACY-1215 combined with IMiDs in cell lines was assessed by measuring 3-(4,5-dimethylthiazol-2-yl)-5-(3-carboxymethoxyphenyl)-2-(4-sulfophenyl)-2H-tetrazolium (MTS; CellTiter 96^®^ AQueous One Solution; Promega; Madison, WI) dye absorbance. Cells from 72 hour cultures were pulsed with 5 μL of CellTiter 96^®^ AQueous One Solution in each well. The 384-well plates were incubated at 37°C for 5 hours, and absorbance was read at a wavelength of 490 nm (with correction using readings at 650 nm) on a spectrophotometer (Molecular Devices Corp.; Sunnyvale, CA). Triplicate values were averaged and SD calculated and graphed with Prism software (GraphPad).

### Synergy analysis

Cells were treated in quadruplicate in 384 well plates with an 8 × 12 dose matrix of ACY-241 or ACY-1215 and each IMiD. After incubating treated cells for 72 hours, total cell viability was assessed via MTS assay. The mean Fraction Affected (F_A_) was established for each dose combination, and based on each single agent dose-effect plot the relative Combination Index (CI) value was determined using CalcuSyn Software (BioSoft; Cambridge, UK) according to the method of Chou and Talalay [33]. The resulting dataset was used to generate FA-CI plots to identify synergistic drug interactions. CI values greater than 2 are indicative of antagonism, CI = 1 represents additivity, and CI < 1 represents synergistic interaction of two agents.

### Apoptosis assay

Cells were treated with the indicated concentrations of compounds. At 96 hours post treatment, cells were harvested, washed twice in PBS, and stained for Annexin V and propidium iodide using the Alexa Fluor 488 Annexin V/Dead Cell Apoptosis Kit (Life Technologies, Grand Island, NY) according to the manufacturer’s protocol. Cells were analyzed on a FC500 flow cytometer (Becton Dickenson). Flow cytometry data were analyzed using FlowJo software and gating results were analyzed and graphed in Prism.

### Cell cycle assay

Cells were treated with the indicated concentrations of compounds. At 72 hours post treatment, cells were incubated for 60 min in the presence of 10 μM 5-ethynyl-2’-deoxyuridine (EdU) at 37°C. After EdU incorporation, cells were washed in PBS and resuspended in fixative solution using the Click-iT EdU Alexa Fluor 488 Flow Cytometry Assay Kit (Life Technologies) and stained according to the manufacturer’s protocol. Cellular DNA was stained using FxCycle^™^ Far Red Stain (Life Technologies) and cells analyzed on an Attune NxT Flow cytometer (ThermoFisher Scientific). Flow cytometry data were analyzed using FlowJo software and gating results were analyzed and graphed in Prism.

### Real time PCR

Total RNA was isolated using RNeasy Micro Kit (Qiagen) with on-column DNase digestion, converted into cDNA using the High Capacity RNA-to-cDNA Kit (Life Technologies), diluted 5-fold with water, and 1.5 μL used as a template for Taqman PCR using Taqman Universal Master Mix II, No UNG (ThermoFisher Scientific) and run on an Applied Biosystems QuantStudio 7 Flex Real-Time PCR System. Relative expression levels were determined by normalization to the housekeeping gene *RPLP1* using the comparative Ct (2^ΔΔCt^) method. Probes used for Taqman: *BIRC5* (Hs00977611_g1), *CRBN* (Hs00372271_m1), *Myc* (Hs00153408_m1), *IRF4* (Hs01056535_m1), *IKZF1* (Hs00958473_m1), *IKZF3* (Hs00918013_m1), *RPLP1* (Hs01653088_g1).

### Animal studies

All animal studies were performed in female CB.17 SCID mice (CB17/Icr-Prkdc^scid^/IcrIcoCrl) at Charles River Discovery Services (CR DS-NC; Morrisville, NC). The identity of H929 cell line was validated and documented by Charles River Discovery Services. The study was approved by the CR DS-NC Institutional Animal Care and Use Committee. This site has an approved Assurance Statement (A4358-01) on file with the Office of Laboratory Animal Welfare (OLAW), National Institutes of Health (NIH) and is accredited by the Association for Assessment and Accreditation of Laboratory Animal Care International (AAALAC International). Animals were obtained from an approved vendor and were housed under conditions which met the requirements specified in the *Guide for the Care and Use of Laboratory Animals* from the National Research Council. The health and welfare of all animals was assessed on a daily basis and animals observed with abnormal clinical signs were brought to the attention of the veterinary staff for monitoring and recommendation of appropriate treatment, including supportive care. Based on veterinary recommendation, animals experiencing severe or chronic pain or distress that could not be relieved were humanely euthanized. Animals on this study were euthanized by exposure to an overdose of isoflurane consistent with the AVMA Guidelines of Euthanasia for the Euthanasia of Animals: 2013 Edition.

IACUC approved general humane end points for tumor models that were applied to this study included: 1) Tumor volume reaching 2000 mm^3^, 2) Any individual animal with a single observation of more than 30% body weight loss or three consecutive measurements of more than 25% body weight loss, and 3) Any group with a mean body weight loss of more than 20% or more than 10% mortality will stop dosing. The group is not euthanized and recovery is allowed. Within a group with more than 20% weight loss, individuals hitting the individual body weight loss endpoint will be euthanized. If the group treatment related body weight loss is recovered to within 10% of the original weights, dosing may resume at a lower dose or less frequent dosing schedule. Exceptions to non-treatment body weight percent recovery may be allowed on a case-by-case basis based on veterinary approval in consultation with the Study Director.

Xenografts were initiated by subcutaneously injecting 100 μL of H929 MM cell suspension (1 x 10^7^ cells) into the right flank of 7-week-old mice. Tumors for all mice were allowed to attain a volume of 100 to 150 mm^3^ before randomization (cohort sizes n = 10) and treatment initiation. ACY-241 was dosed via intraperitoneal (IP) injection at 50 mg/kg once daily for 42 consecutive days, while pomalidomide was dosed via IP injection at 1 mg/kg once daily for 42 consecutive days. Tumor volumes and body weights were measured twice weekly throughout the duration of the study, and mean ± SD are plotted for each group up to the timepoint when the first animal in each cohort was sacrificed for humane or tumor volume endpoints. Differences in tumor volume are indicated by *p* values obtained by performing a t-test at the indicated tumor measurement time points.

## Supporting information

S1 Fig ACY-241 and ACY-1215 exhibit similar effects on cell proliferation and viability. A) H929 cells were treated with ACY-1215 and live cells were counted following trypan blue staining. B) H929 cells were treated with increasing concentrations of either ACY-241 or ACY-1215 for 3 days and cell viability was measured using an MTS assay. The IC_50_ was determined for each treatment condition. C) Jeko-1 cells were treated with increasing concentrations of ACY-241 for 4 days and cell viability was measured using an MTS assay and the IC_50_ calculated.
(TIF)

S2 Fig ACY-1215 synergizes with lenalidomide and pomalidomide to suppress viability of MM cells. A) Jeko-1 cells were treated with increasing concentrations of ACY-241 and either lenalidomide or pomalidomide in an escalating concentration matrix. Cells were incubated for 3 days followed by measuring cell viability by MTS assay. CI values were calculated, with a CI value <1 indicating synergistic activity of the combination over single agent treatment. B) H929 and MM.1s cell lines were treated with increasing concentrations of ACY-1215 and either lenalidomide or pomalidomide in an escalating concentration matrix. Cells were incubated, assayed and analyzed as in (A). Data shown is representative of three independent experiments in each cell line.
(TIF)

S3 Fig Combination treatment with ACY-241 and pomalidomide in Jeko-1 cells. A) Jeko-1 cells were treated with 1 μM pomalidomide or 3 μM ACY-241 alone or in combination for 4 days followed by staining for Annexin V/PI to measure apoptosis. Percent apoptosis was assessed by double positivity for Annexin V/PI. Representative flow dot plots are shown for each cell line (*left*) and the mean ± SD of three independent experiments is plotted (*right*). *** *p* < 0.001. B) Cells treated as in A) were harvested after 48 hours. Total protein was isolated and probed with antibodies for Survivin, Caspase 3, Cleaved Caspase 3, and β-Actin. Results are representative of at least 3 independent experiments for each antibody. C) Jeko-1 cells treated as in A) were harvested after 48 hours. Total RNA was isolated and converted to cDNA followed by real-time PCR for *BIRC5* (survivin). Results were normalized to the housekeeping gene *RPLP1*, and the mean ± SD of three independent experiments is shown. D) Jeko-1 cells were treated as in A) for 3 days followed by incubation for 1 hour with EdU and stained for EdU incorporation and FxCycle Far Red to measure S phase frequency. Percent cells in S phase was determined by gating EdU positive cells. Representative flow dot plots are shown for each cell line (*left*) and the mean ± SD or three independent experiments is plotted (*right*). *** *p* < 0.001. E) Jeko-1 cells treated as in A) were harvested after 48 hours. Total protein was isolated and probed with antibodies for Myc, IRF4, IKZF3 and β-Actin. Results are representative of at least 3 independent experiments for each antibody.
(TIF)

S4 Fig Relative gene expression changes in response to ACY-241 and/or pomalidomide treatment. A) H929, U266, MM.1s, and Jeko-1 cells were treated with 1 μM or 0.05 μM pomalidomide or 3 μM ACY-241 alone or in combination and harvested after 48 hours. Total RNA was isolated and converted to cDNA followed by real-time PCR for Cereblon (CRBN), Myc, IRF4, IKZF1, and IKZF3. Results were normalized to the housekeeping gene *RPLP1* and the mean ± SD of triplicate samples was determined. Data shown is representative of at least three independent experiments.
(TIF)

S5 Fig Combination treatment with ACY-241 and either lenalidomide or pomalidomide results in decreased expression of survival factors and increased expression of apoptotic markers. A) H929 cells were treated with 2 μM lenalidomide (Len), 1 μM pomalidomide (Pom) and/or 3 μM ACY-241. Cells were harvested after 48 hours and total protein was isolated and probed with antibodies for Myc, IRF4, IKZF3, and β-Actin. B) MM.1s cells treated as in A) were harvested after 48 hours and total protein was isolated and probed with antibodies for Myc, IRF4, IKZF3, Caspase 3, and β-Actin.
(TIF)
