# Supplementary figures and images for: Enhancement of pomalidomide anti-tumor response with ACY-241, a selective HDAC6 inhibitor
Source: PLoS One. 2017 Mar 6;12(3):e0173507. doi: 10.1371/journal.pone.0173507 (PMC5338861; doi:10.1371/journal.pone.0173507)

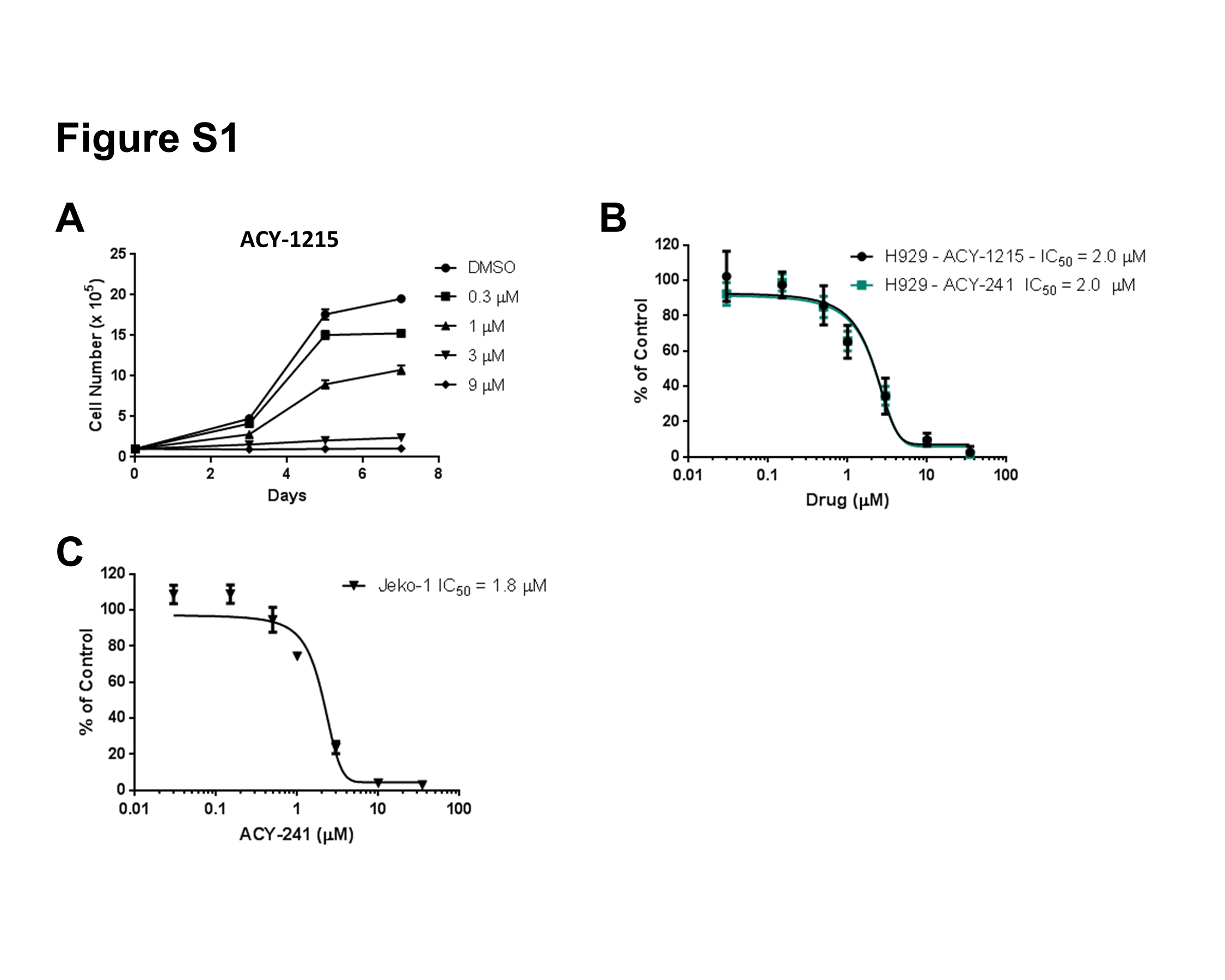

Supplement: S1 Fig — A) H929 cells were treated with ACY-1215 and live cells were counted following trypan blue staining. B) H929 cells were treated with increasing concentrations of either ACY-241 or ACY-1215 for 3 days and cell viability was measured using an MTS assay. The IC50 was determined for each treatment condition. C) Jeko-1 cells were treated with increasing concentrations of ACY-241 for 4 days and cell viability was measured using an MTS assay and the IC50 calculated. (TIF) [file pone.0173507.s001.tif]

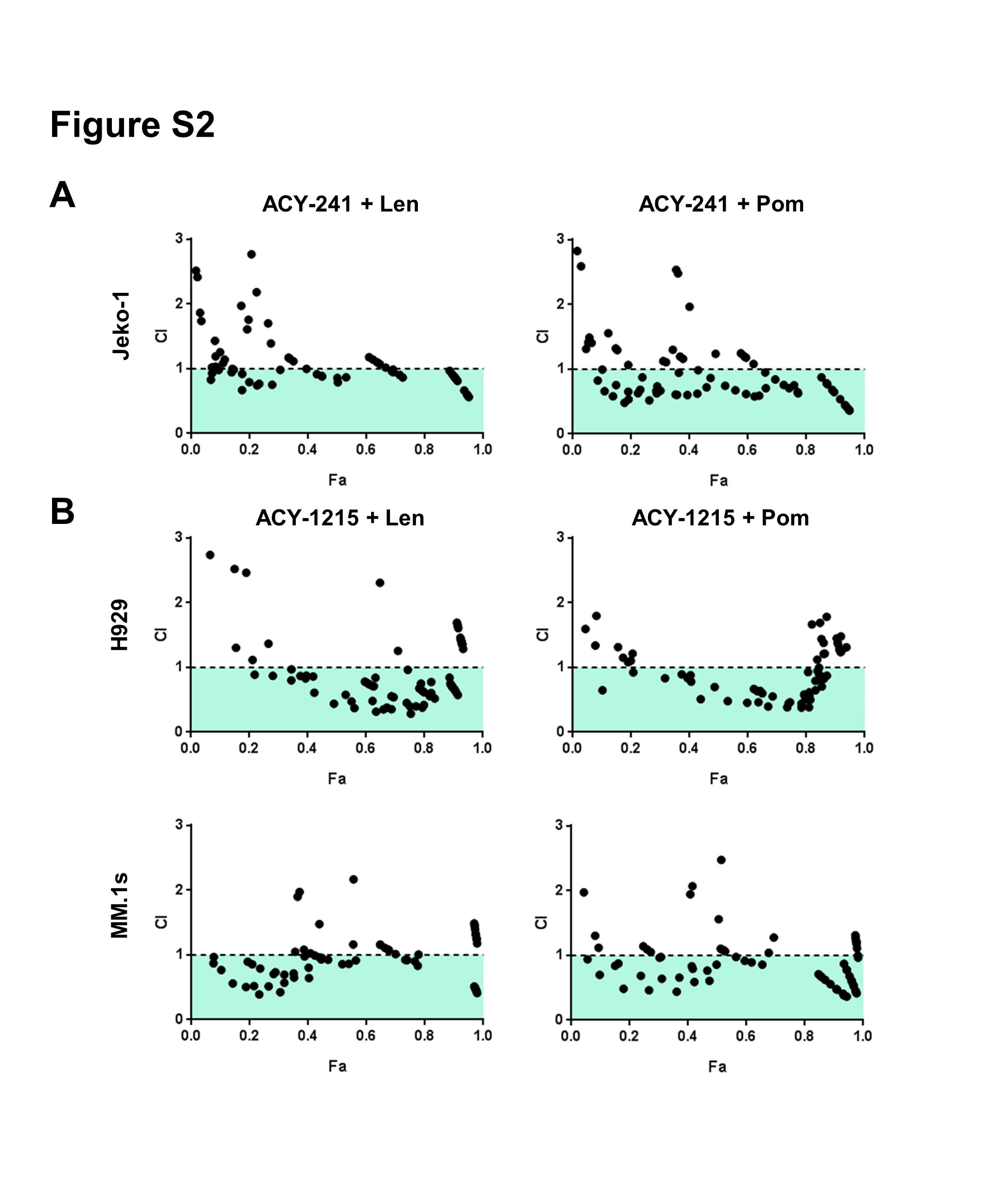

Supplement: S2 Fig — A) Jeko-1 cells were treated with increasing concentrations of ACY-241 and either lenalidomide or pomalidomide in an escalating concentration matrix. Cells were incubated for 3 days followed by measuring cell viability by MTS assay. CI values were calculated, with a CI value <1 indicating synergistic activity of the combination over single agent treatment. B) H929 and MM.1s cell lines were treated with increasing concentrations of ACY-1215 and either lenalidomide or pomalidomide in an escalating concentration matrix. Cells were incubated, assayed and analyzed as in (A). Data shown is representative of three independent experiments in each cell line. (TIF) [file pone.0173507.s002.tif]

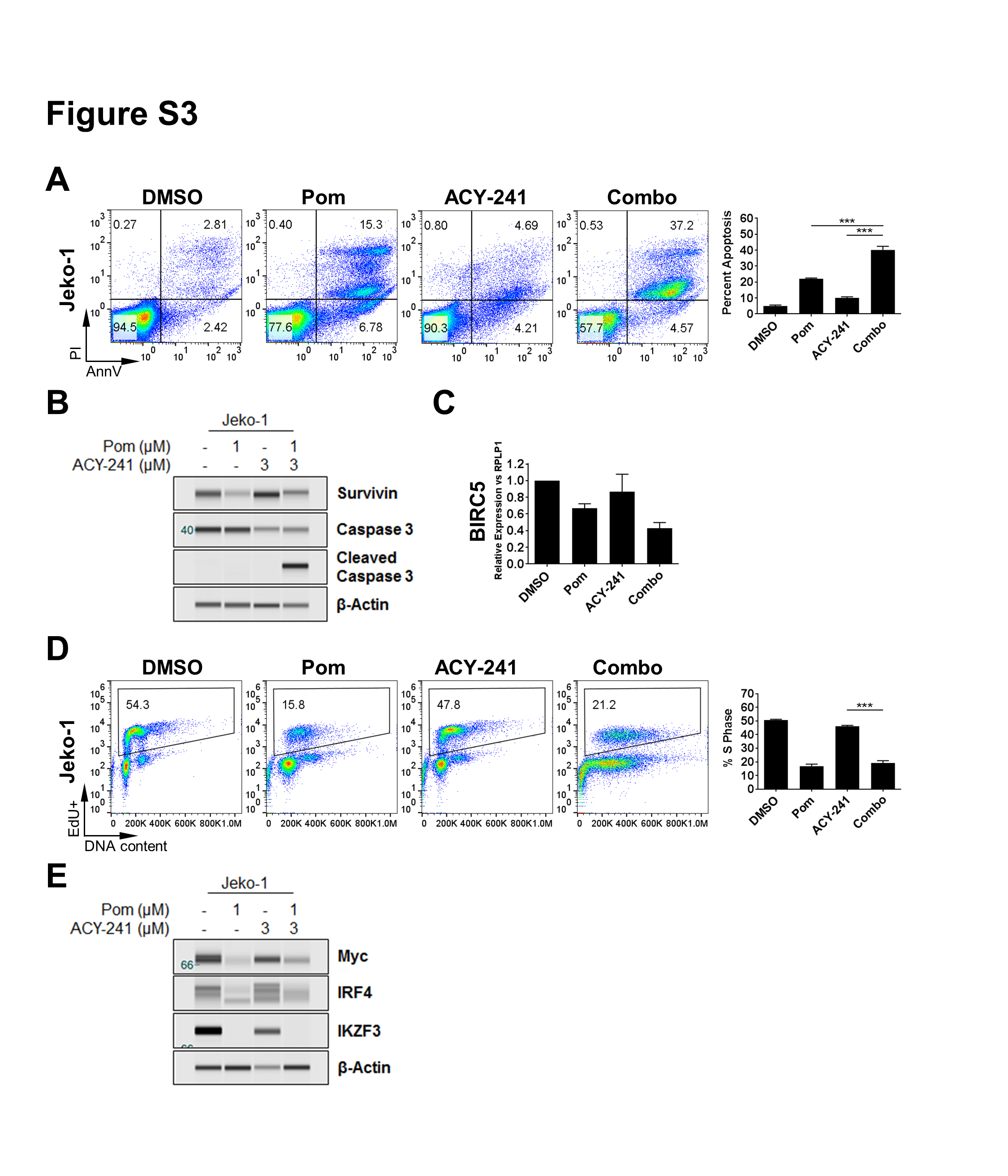

Supplement: S3 Fig — A) Jeko-1 cells were treated with 1 μM pomalidomide or 3 μM ACY-241 alone or in combination for 4 days followed by staining for Annexin V/PI to measure apoptosis. Percent apoptosis was assessed by double positivity for Annexin V/PI. Representative flow dot plots are shown for each cell line (left) and the mean ± SD of three independent experiments is plotted (right). *** p < 0.001. B) Cells treated as in A) were harvested after 48 hours. Total protein was isolated and probed with antibodies for Survivin, Caspase 3, Cleaved Caspase 3, and β-Actin. Results are representative of at least 3 independent experiments for each antibody. C) Jeko-1 cells treated as in A) were harvested after 48 hours. Total RNA was isolated and converted to cDNA followed by real-time PCR for BIRC5 (survivin). Results were normalized to the housekeeping gene RPLP1, and the mean ± SD of three independent experiments is shown. D) Jeko-1 cells were treated as in A) for 3 days followed by incubation for 1 hour with EdU and stained for EdU incorporation and FxCycle Far Red to measure S phase frequency. Percent cells in S phase was determined by gating EdU positive cells. Representative flow dot plots are shown for each cell line (left) and the mean ± SD or three independent experiments is plotted (right). *** p < 0.001. E) Jeko-1 cells treated as in A) were harvested after 48 hours. Total protein was isolated and probed with antibodies for Myc, IRF4, IKZF3 and β-Actin. Results are representative of at least 3 independent experiments for each antibody. (TIF) [file pone.0173507.s003.tif]

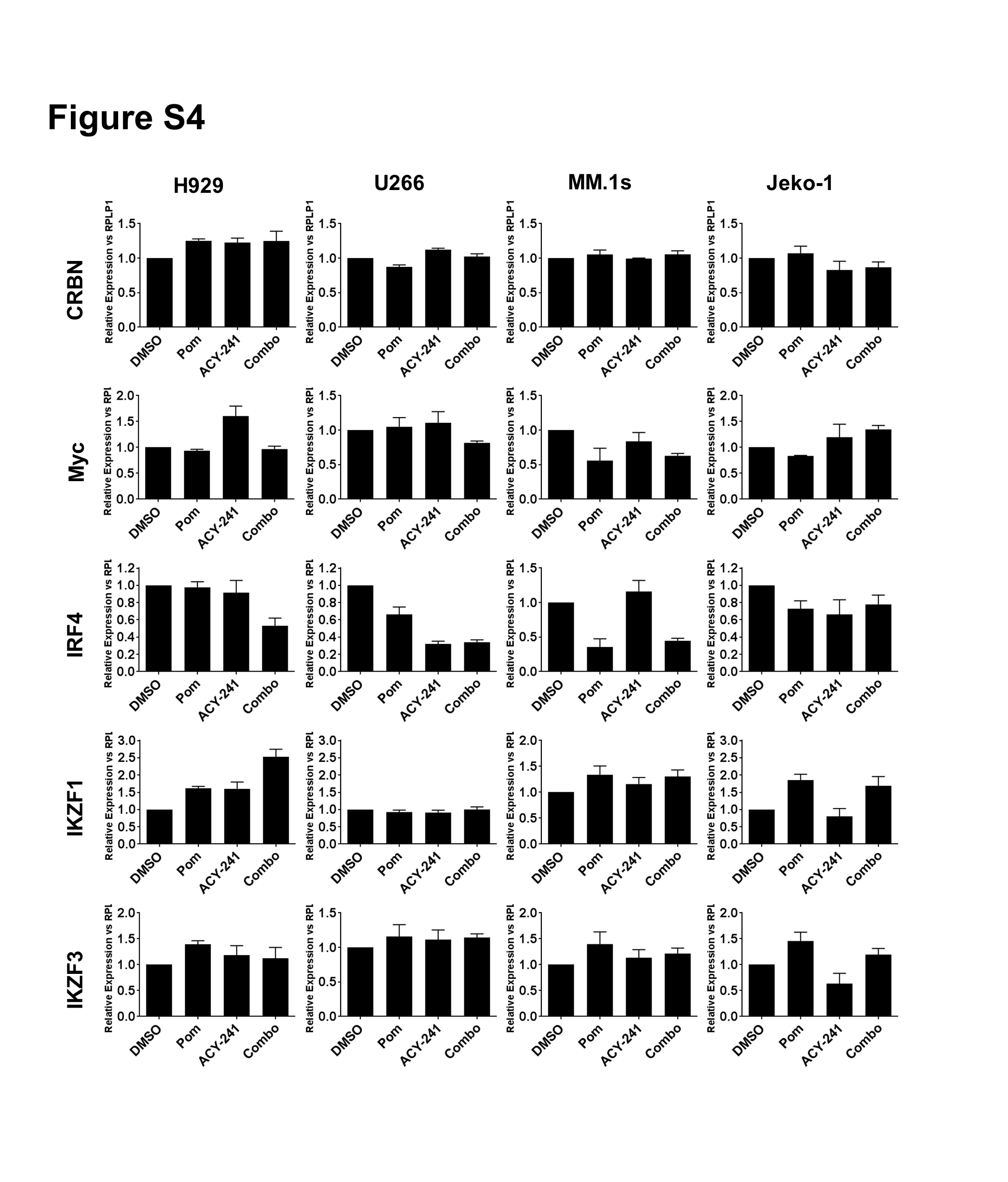

Supplement: S4 Fig — A) H929, U266, MM.1s, and Jeko-1 cells were treated with 1 μM or 0.05 μM pomalidomide or 3 μM ACY-241 alone or in combination and harvested after 48 hours. Total RNA was isolated and converted to cDNA followed by real-time PCR for Cereblon (CRBN), Myc, IRF4, IKZF1, and IKZF3. Results were normalized to the housekeeping gene RPLP1 and the mean ± SD of triplicate samples was determined. Data shown is representative of at least three independent experiments. (TIF) [file pone.0173507.s004.tif]

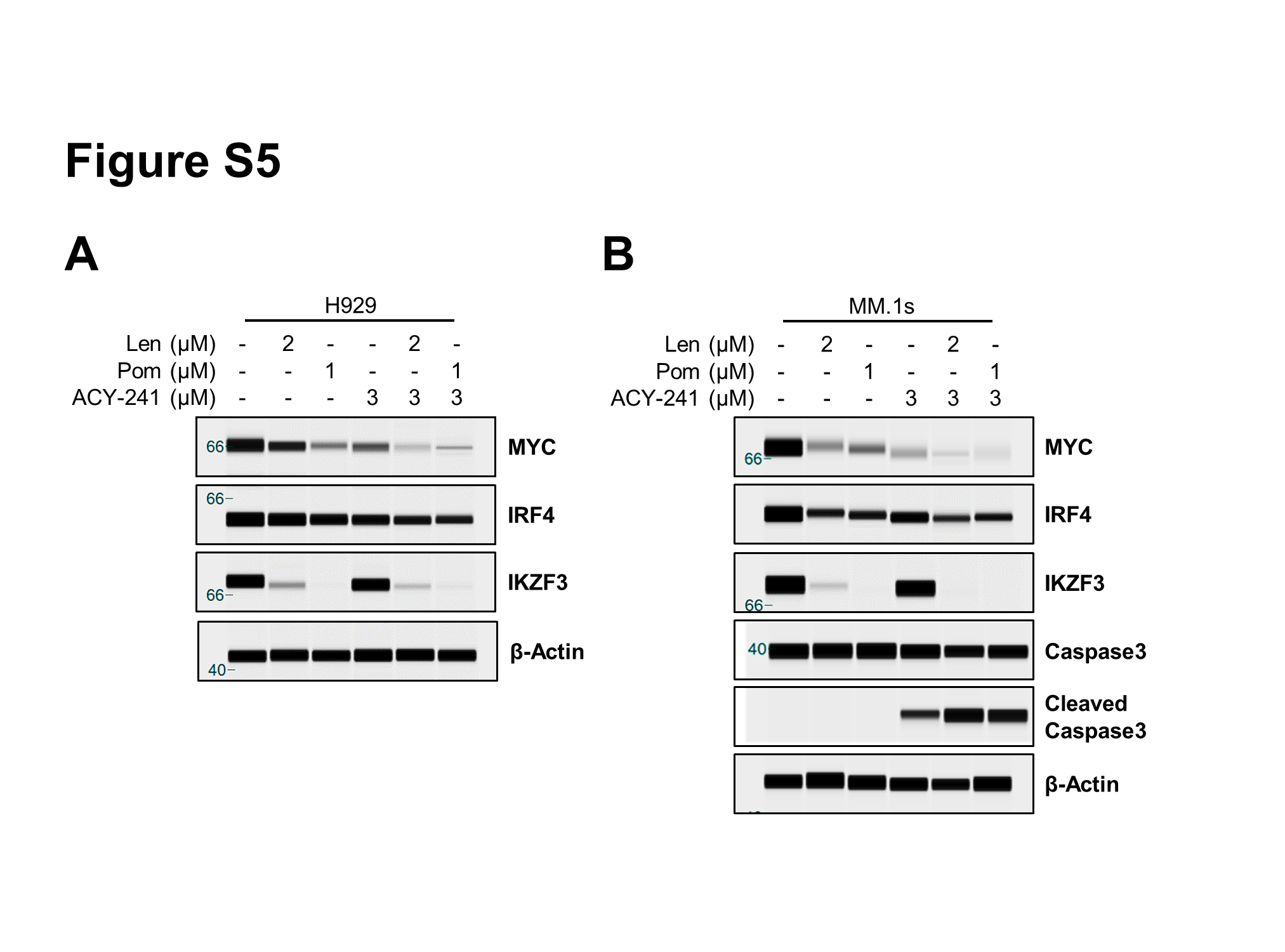

Supplement: S5 Fig — A) H929 cells were treated with 2 μM lenalidomide (Len), 1 μM pomalidomide (Pom) and/or 3 μM ACY-241. Cells were harvested after 48 hours and total protein was isolated and probed with antibodies for Myc, IRF4, IKZF3, and β-Actin. B) MM.1s cells treated as in A) were harvested after 48 hours and total protein was isolated and probed with antibodies for Myc, IRF4, IKZF3, Caspase 3, and β-Actin. (TIF) [file pone.0173507.s005.tif]
